# Exploring the relationship between Ayurgenomics and bio-psycho-social symptoms in cancer care

**DOI:** 10.1016/j.jaim.2026.101321

**Published:** 2026-05-21

**Authors:** Padmashanti Nilachal, Ananda Balayogi Bhavanani, Uthpala Gajamanne, Vandana Srivastava, Karthikeyan Kaliyamurthi

**Affiliations:** a Institute of Salutogenesis & Complementary Medicine (ISCM), Sri Balaji Vidyapeeth (Deemed-to-be University), Pondicherry, India; b Department of Ayurveda and Holistic Health, Dev Sanskriti Vishwavidyalaya, Gayatrikunj-Shantikunj, Haridwar, Uttarakhand, 249411, India; c Department of Medical Oncology, MGMC & RI Hospital, Sri Balaji Vidyapeeth (Deemed to be University), Pondicherry, India

**Keywords:** Cancer, Ayurgenomics, AyuSoft, Integrated medicine, Oncology, Karkatabudh, Yoga therapy

## Abstract

**Background:**

Ayurveda, the ancient Indian system of medicine, emphasises the role of individual constitution in health. Modern Ayurgenomics integrates Ayurvedic principles with genomic analysis, aiming to personalize Yogic interventions for cancer patients.

**Objective:**

The current study aims to explore the intersection of Ayurgenomics, *dehaprakriti*, and Yoga Therapy in the context of medical oncology.

**Method:**

The study included 60 chemotherapy patients, with their *dehaprakriti* assessed using AyuSoft. Patients' symptoms were diagnosed with the Rotterdam Symptom Checklist (RSCL). The data was analyzed to observe any correlation between Prakriti type and chemotherapy response.

**Results:**

*Vatapradhan* types show high physical impairment (38.96) but retain functionality (13.83). *Pittapradhan* types have minimal physical burden (24.05) but high psychological strain (13.00) with a positive life outlook. *Kapha*-dominant types experience moderate physical (27.78–34.61) and psychological burdens (9.69–12.12) and slower disease progression. Major findings revealed that *vata*-dominant patients required more immediate intervention due to rapid tumor growth, while *pitta*-dominant patients were more susceptible to chemotherapy side effects. Patients with *kaphaprakriti* showed slower tumor growth. Appropriate Yogic interventions and dietary adjustments may help manage the loss of vitality during chemotherapy.

**Conclusion:**

The study highlights the growing relevance of integrating traditional Eastern wisdom, such as *Ayurveda*, with Western scientific approaches to personalize cancer care. Integrating Prakriti-based Yogic interventions may alleviate the physical and psychological impact of cancer and its treatments, enhancing the overall quality of life for patients.

## 1 Introduction

### 1.1 Background

**Fig. 1:**
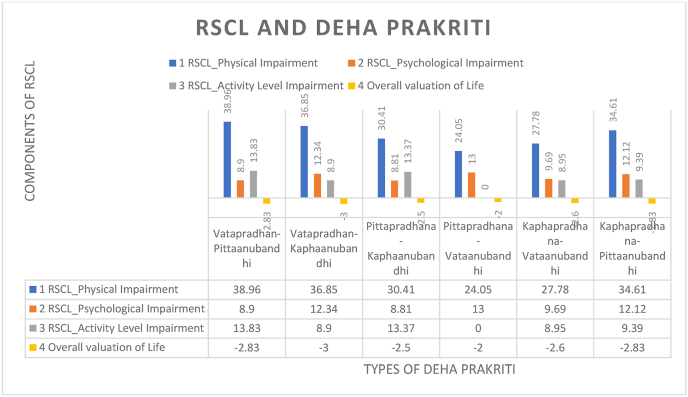
Relationship between **RSCL and Deha prakriti analysis**.

As defined by National Cancer Institute, “Cancer is a disease in which some of the body's cells grow uncontrollably and spread to other parts of the body” [1]. Cancers are a large and heterogeneous group of malignant tumors that collectively accounted for approximately 600,000 US deaths in 2020 [2]. It is estimated that currently, there are about 1.3 million new cancer cases and approximately 0.85 million deaths due to cancer every year, and these are estimated to increase to about 2.1 million and 1.3 million, respectively, by 2040 globally [3] (see Table 1, Fig. 1)

**Table 1:** Mean scores of Rotterdam Symptom Checklist (RSCL) parameters.

RSCL Parameter	*Vatapradhana*-*Pittaanubandhi*	*Vatapradhana-Kaphaanubandhi*	*Pittapradhana-Kaphaanubandhi*	*Pittapradhana-Vataanubandhi*	*Kaphapradhana-Vataanubandhi*	*Kaphapradhana-Pittanubandhi*
RSCL Physical Impairement	38.96	36.85	30.41	24.05	27.78	34.61
RSCL Psychological Impairement	8.90	12.34	8.81	13.00	9.69	12.12
RSCL Activity Level Impairement	13.83	8.90	13.37	0.00	8.95	9.39
Overall Valuation of Life	−2.83	−3.00	−2.50	−2.00	−2.60	−2.83

In India as well, the condition is not so good. Cancer in India presents unique epidemiological and public health challenges, influenced by environmental, socioeconomic, and lifestyle factors [[4], [5], [6]]. India experiences a varied incidence of cancer types, with breast, cervical, oral, and lung cancers being the most prevalent [7]. The regional distribution of cancers is diverse, with a high rate of cervical cancer in rural areas due to limited access to early screening and vaccinations [8,9]. Breast cancer rates are increasing due to urbanization, lifestyle changes, and delayed childbirth​ [10]. The cancer burden in India continues to rise, with an estimated 1.39 million new cases reported in 2020 [8]. This figure is projected to increase to 1.6 million by 2025 [11]. The most prevalent cancer sites include the breast, lung, mouth, cervix uteri, and tongue. Cancer mortality remains significant, with approximately 700,000 deaths reported in 2012 [12,13]. Notably, oral cavity and lung cancers in males, along with cervix and breast cancers in females, account for over 50 % of all cancer deaths [13,14]. Furthermore, a substantial proportion of patients are diagnosed at locally advanced stages, such as breast, cervix uteri, head and neck, and stomach cancers, while lung cancer often presents with distant metastasis [14].

Cancer is a multifaceted process of proliferation that permeates various aspects of the human experience. It manifests not only in the uncontrolled growth of cells, but also in the proliferation of suffering, the expansion of medical interventions, the advancement of technology, and the ever-evolving narratives that surround it. This disease touches the very core of our existence, profoundly impacting individuals, families, communities, and entire nations, often in deeply personal and far-reaching ways [15].

In such a scenario, Indian Knowledge System (IKS) has a lot to offer. The ancient Indian knowledge of Ayurveda and Yoga may play a very important role in the field of oncology [16]. Recent advancements in the field of Ayurgenomics, which combines Ayurvedic principles with genomic analysis, have shown promise in personalizing Yogic interventions for cancer patients. Also, Yoga, another ancient Indian practice, has gained recognition for its potential benefits in supporting the well-being of cancer patients [17].

Ayurveda emphasises the role of individual constitution in health and disease [18,19]. Ayurveda focuses on whole-person approach, integrating anatomical, physiological, and psychological aspects to address an individual's unique dosha, which shapes their character and overall well-being [20]. The Tridosha theory in Ayurveda postulates that there are three fundamental pathophysiological entities, known as doshas, which govern the unique constitution of each individual and forms the basis for all body function [21]. These doshas - *Vata*, *Pitta*, and *Kapha* - each possess their own distinct characteristics and functions that collectively shape the individual's anatomical, physiological, and psychological attributes [21].

The *vata* dosha is responsible for various physiological processes within the body, including the transmission of molecules to nerve impulses [22]. It contributes to the manifestation of form, cell division, signaling, waste elimination, movement, and thought processes [22,23]. Additionally, *vata* helps to regulate the functions of the *pitta* and *kapha* *doshas* [24,25]. *pitta* governs digestive and metabolic processes at the cellular level [23]. It also plays a crucial role in regulating energy homeostasis, temperature control, visual function, pigmentation, and immune surveillance, among other physiological functions [26,27]. The *kapha* *dosha* is responsible for maintaining the body's structural integrity and cohesiveness [28]. It governs processes such as storage, stability, development, and physiological maintenance [29]. Additionally, *kapha* provides lubrication to the joints, mucous membranes, and other bodily tissues [30].

Ayurvedic principles define Dosha Prakriti as the unique physical constitution of an individual, which encompasses their physical, physiological, psychological, and spiritual attributes. This constitution is determined at the time of conception and remains unchanged throughout one's lifetime. Prakriti is composed of varying combinations of the three doshas - *Vata*, *Pitta*, and *Kapha* - present at the time of conception.

Individuals can have different dominant doshas, which explains why people react differently to similar situations. According to Ayurveda, each person has a distinct body constitution that is unique and different from others. Therefore, individuals should follow specific dietary and lifestyle practices tailored to their Prakriti for disease prevention and overall health maintenance. Assessing an individual's Prakriti is valuable for physicians in predicting disease response, determining the course of the disease, making prognosis judgments, and designing appropriate therapeutic regimens. This Prakriti is further categorized into two types: Deha Prakriti and Manasa Prakriti. Deha Prakriti is classified into 7 subtypes - 3 Ekadoshaja, 3 Dwidoshaja/Samsargaja, and 1 Tridoshaja/Sannipataja [31]. Individuals with different Deha Prakriti possess variations in both their psychological and physical characteristics.

These three Doshas interact dynamically, highlighting their essential role in Ayurvedic health principles. This interaction forms the foundation of Deha Prakriti, or individual constitution, which represents the unique balance of Doshas within each person [23]. Prakriti shapes one's physical, physiological, and psychological characteristics, influencing their health profile and susceptibility to various conditions, including cancer. Ayurveda suggests that a personalized approach to cancer management, based on the individual's Prakriti, can lead to more effective and tailored interventions [16]. Ayurveda has a long history of using natural drugs and treatments to prevent or suppress various types of tumors. Ayurvedic texts categorize cancer as either inflammatory or non-inflammatory swellings. The classical Ayurvedic texts describe the disease that exhibits symptoms similar to malignancy as Arbuda [[32], [33], [34], [35]].

The Sushruta Samhita provides detailed and precise treatment protocols for Arbuda, while the Ashtanga Hridaya offers similar treatment approaches. Ayurvedic texts, such as Madanapala Nighantu, have also documented various chemical treatments using Rasa Aushadhi for Arbuda, dating back to 800-1400 AD [[35], [36], [37]]. Ayurgenomics not only enhances the personalization of cancer therapies through genomic insights but also aligns with holistic Ayurvedic practices that consider an individual's unique constitution.

Ayurvedic texts also describe about Yagya (fire rituals) and its benefits related to cancer [38]. This Yagyopathy approach for the treatment of Arbuda, utilizing specific herbal fumigation and medicated materials to create a controlled environment that supports the healing process [38,39].

Yoga therapy, in parallel, complements these personalized treatments by addressing the physical, emotional, and spiritual needs of cancer patients [40]. This ancient Indian practice can be seamlessly integrated into a comprehensive, personalized cancer care plan, offering a cost-effective and accessible approach to enhancing the quality of life for these patients [41]. Yoga therapy has provided benefits for cancer patients in various ways. Cancer patients undergoing chemotherapy frequently suffer from many side effects. But appropriate yoga practices can help mitigate these, improving their well-being and quality of life [42,43]. Studies have shown yoga can enhance sleep quality, especially for breast cancer patients with chemotherapy-related insomnia [44]. Patients participating in yoga programs have seen significant improvements in measures of sleep, quality of life, stress, fatigue, and mood [41]. Yoga has also been found to increase immune function in cancer patients.

Thus, Ayurgenomics and Yoga together offer a multifaceted approach to cancer care, combining genetic precision with holistic wellness. Modern Ayurgenomics integrates Ayurvedic principles with genomic analysis, aiming to personalize Yogic interventions for cancer patients [45].

### 1.2 Objectives

The current study aims to explore the intersection of Ayurgenomics, Deha Prakriti, and Yoga Therapy in the context of cancer oncology.

1. To explore the intersection of Ayurgenomics, Deha Prakriti, and Yoga Therapy in the context of cancer oncology.
2. To assess the influence of Deha Prakriti on chemotherapy responses and its implications for treatment personalization.

## 2 Methods

### 2.1 Study design

This study examines the relationship between cancer patients undergoing chemotherapy and Deha Prakriti using a cross-sectional observational design. 60 patients receiving chemotherapy were involved in the study, which was carried out in the medical oncology department. Participants were not limited based on their demographics, the type of cancer they had, or the stage of their sickness, guaranteeing a diverse and inclusive sample. Data on Prakriti classification and chemotherapy outcomes were collected at a single point in time, without longitudinal follow-up. Confidentiality and voluntary participation were prioritized throughout the research process. By analyzing the interplay between traditional Ayurvedic principles and modern oncology, this study aims to provide insights into personalized cancer care strategies.

### 2.2 Settings

The study was conducted in the department of medical oncology at Mahatma Gandhi Medical College of Sri Balaji Vidyapeeth (Deemed to be University), Pondicherry. The research took place over a defined period, with patient recruitment occurring between August 2nd^,^ 2024 and November 30th^,^ 2024. Data collection was performed during routine chemotherapy sessions, ensuring minimal disruption to standard patient care. Since this was a cross-sectional study, there was no extended follow-up period, and all relevant data on Prakriti classification and chemotherapy responses were gathered at the time of recruitment.

### 2.3 Participants

The study included 60 patients undergoing chemotherapy in the department of medical oncology. Participants were not restricted based on demographics, cancer types, or disease stages**,** ensuring a diverse and inclusive sample. Eligible participants were required to:

• Be diagnosed with cancer and currently undergoing chemotherapy.
• Provide informed consent for participation.
• Be capable of completing the Rotterdam Symptom Checklist (RSCL) for symptom assessment.

**Exclusion criteria** included patients with severe cognitive impairment or inability to provide informed consent.

### 2.4 Variables

The study examined multiple variables:

#### 2.4.1 Primary exposure (Predictor)

Ayurvedic Prakriti classification, assessed using AyuSoft, a validated diagnostic tool that categorizes individuals into 10 Prakriti types based on dominant *vata*, *pitta*, and *kapha* characteristics.

Deha Prakriti evaluation for all participants was conducted using AyuSoft, an advanced diagnostic tool developed by the Centre for Development of Advanced Computing. AyuSoft assesses anatomical, physiological, and psychological traits to classify individuals into one of 10 combinations of vata, *pitta*, and *kapha* types based on dominant characteristics. This tool enabled standardized and reliable Prakriti classification across the cohort, minimizing variability. As per *Charakaachaarya*, Dosha Prakriti is classified in to seven types - 3 Ekadoshaja, 3 Dwidoshaja/Samsargaja, and 1 Tridoshaja/Sannipataja. Individuals with different Deha Prakriti possess variations in both their psychological and physical characteristics. In view of practical convenience and possibility of *Prakriti* result, AyuSoft has considered ten types of *Prakriti*.

Table 2 shows that in combined *Dosha Prakriti,* dominance of first *Dosha* is greater than later one e.g. *Kapha Pradhaana Pittaanubandhi* (*Kapha* is predominant *Dosha* as compared to *Pitta Dosha*)

**Table 2:** Correlation between Prakriti type and dominant Dosha.

	Type	Dominant Dosha
1.	*Ekaantika Vaata Prakriti*	*Vaata*
2	*Ekaantika Pitta Prakriti*	*Pitta*
3	*Ekaantika Kapha Prakriti*	*Kapha*
4	*Kaphapradhaana Pittaanubandhi Prakriti*	*Kapha Pitta*
5	*Kaphapradhaana Vaataanubandhi Prakriti*	*Kapha Vaata*
6	*Pittapradhaana Kaphaanubandhi Prakriti*	*Pitta Kapha*
7	*Pittapradhaana Vaataanubandhi Prakriti*	*Pitta Vaata*
8	*Vaatapradhaana Kaphanubandhi Prakriti*	*Vaata Kapha*
9	*Vaatapradhaana Pittaanubandhi Prakriti*	*Vaata Pitta*
10	*Sama Prakriti*	All three *Dosha* are in a balanced state. This is considered as ideal but very rare condition.

#### 2.4.2 Validation and reliability of the AyuSoft tool

AyuSoft constitutes a computer-assisted diagnostic platform developed by the Centre for Development of Advanced Computing, India, in partnership with the Department of AYUSH and multiple Ayurvedic institutions. The software incorporates classical Ayurvedic tenets from texts such as *Charaka Samhita*, *Sushruta Samhita*, and *Ashtanga Hridaya* into an algorithmic framework that systematically assesses anatomical, physiological, and psychological attributes to delineate an individual's Deha Prakriti profile.

The validity and reliability of AyuSoft have been established across several independent studies [46]. Earlier validations demonstrated robust inter-rater and test-retest reliability relative to expert Ayurvedic evaluations in both healthy volunteers and clinical cohorts [47,48].

Although dedicated validation within oncology populations is limited, the tool has been effectively applied in integrative medicine research on chronic and metabolic disorders, yielding objective and reproducible Prakriti classifications.

In this study, *Deha Prakriti* assessment using AyuSoft was conducted by the Principal Investigator (PI), who is trained in the use of the software, with support from an Ayurveda physician experienced in clinical *Prakriti* evaluation.

#### 2.4.3 Primary outcome

Symptom burden prior to chemotherapy, measured using the Rotterdam Symptom Checklist (RSCL) across four domains:

• Physical symptoms (fatigue, pain, nausea, etc.)
• Psychological well-being (anxiety, depression, emotional distress)
• Activity level (functional limitations)
• Life valuation (sense of purpose and satisfaction)

To explore the integrative relationship between symptom burden and Ayurvedic constitution, the RSCL domains were also interpreted in terms of Ayurvedic constructs of *Tridosha* and *Deha Prakriti*, as detailed below:

The Rotterdam Symptom Checklist (RSCL) is a comprehensive and validated instrument that assesses the multifaceted impacts of illness and treatment, particularly in cancer patients. It is a self-report measure that evaluates four key domains: physical health, psychological well-being, activity level, and overall life valuation. The physical health domain of the RSCL identifies symptom burdens like fatigue, pain, and nausea, enabling targeted interventions. Regarding psychological well-being, it captures emotional distress, anxiety, and depression, providing insights into the mental health challenges faced by patients. The focus on activity level helps track functional limitations, revealing how illness or treatment affects daily activities and independence. The life valuation domain assesses the individual's subjective sense of purpose and satisfaction, highlighting the broader existential impacts of the illness. By comprehensively addressing these areas, the RSCL plays a vital role in personalized care planning, monitoring quality of life over time, and evaluating the efficacy of medical or psychosocial interventions. Prior to chemotherapy, participants completed the checklist, providing a comprehensive record of their pre-treatment symptom burden and overall health status. This baseline dataset formed the foundation for analyzing the relationship between Prakriti types and pre-treatment symptom patterns.

Ayurvedic Interpretation of RSCL Domains.

To integrate the Rotterdam Symptom Checklist with Ayurvedic principles, the symptom domains were analyzed in relation to Tridosha equilibrium and variations in Deha Prakriti.

• The Physical Symptom Domain indicates disruptions in *vata* *dosha* (governing motion, neural activity, and fatigue), *pitta* *dosha* (regulating metabolism, inflammation, and thermoregulation), and *kapha* *dosha* (overseeing structure, stability, and fluid retention). Specific examples include fatigue and pain linked to Vata aggravation, nausea and heat sensitivity to *pitta* excess, and heaviness or edema to *kapha* disturbance.
• The Psychological Well-being domain corresponds to Manasika Prakriti and Dosha-mediated emotional states, with *vata* associated with anxiety and fear, *pitta* with irritability and frustration, and *kapha* with detachment and sluggishness.
• Activity Level relates to the expressions of Prana Vayu, Tejas, and Ojas; excessive *vata* may hinder function through restlessness and exhaustion, while elevated *kapha* can impair motivation and stamina.
• Life Valuation mirrors Ayurvedic notions of *sattva* and *bala*, wherein balanced *Tridosha* and robust *sattva* correlate with greater life satisfaction, and excesses of *rajas* or *tamas* align with reduced optimism and vitality.

Accordingly, the RSCL domains were evaluated not only as somatic and psychological measures but as indicators of Dosha predominance and dysregulation. This synthesis offers a conceptual model for interpreting symptom variability in cancer patients across *deha* *prakriti* categories, justifying individualized *Ayurvedic* or *Yogic* interventions.

#### 2.4.4 Potential confounders and effect modifiers

Age, sex, cancer type, and stage, as these factors may influence symptom severity and chemotherapy responses.

### 2.5 Measurement

#### 2.5.1 Prakriti classification

Conducted using AyuSoft, which systematically evaluates anatomical, physiological, and psychological traits to ensure standardized classification.

#### 2.5.2 Symptom assessment

The Rotterdam Symptom Checklist was administered in participants' preferred language. Most completed it independently; for those with literacy or language barriers, the Principal Investigator or staff read items verbatim in the native language and recorded responses per RSCL guidelines.

#### 2.5.3 Data collection timing

Since this is a cross-sectional study, all assessments were conducted at a single time point, before chemotherapy administration.

#### 2.5.4 Group comparability

The use of AyuSoft for Prakriti assessment and a validated self-reported RSCL ensured consistency across participants.

### 2.6 Bias

To minimize selection bias, inclusion criteria were broad, allowing a diverse patient population. Measurement bias was reduced by using AyuSoft for Prakriti classification, ensuring standardized assessments. Self-report bias in symptom assessment was addressed by using the validated RSCL questionnaire, which captures a comprehensive and multidimensional symptom profile.

### 2.7 Study size

The sample size of 60 patients was determined chiefly by the feasibility of participant recruitment within the designated study period and the resources available in the oncology department. As an exploratory cross-sectional investigation intended to discern potential associations between Deha Prakriti and symptom burden, a post-hoc power analysis was undertaken to appraise the suitability of this sample size.

Utilizing the observed effect sizes between Prakriti types and Rotterdam Symptom Checklist scores, at a significance level of 0.05, the cohort of 60 participants afforded approximately 80 % statistical power to detect moderate correlations. This substantiates the adequacy of the sample for exploratory inferential analyses.

Subsequent investigations employing larger, stratified samples across cancer types and stages are recommended to corroborate and broaden these preliminary observations.

### 2.8 Quantitative variables

2.8.1. Prakriti types were treated as categorical variables, with ten possible classifications.
2.8.2. Symptom scores from the RSCL were analyzed as continuous and categorical variables, with severity levels grouped for comparative analysis.
2.8.3. Chi-square tests and cross-tabulations were used to examine symptom distributions across different Prakriti types.

### 2.9 Statistical methods

Descriptive statistics were used to summarize patient demographics, *Deha Prakriti* distributions, and baseline symptom burden. Categorical variables were expressed as frequencies and percentages, while continuous variables were summarized as mean ± standard deviation (SD).

Initial associations between *Prakriti* classifications and symptom severity were explored using Chi-square tests and cross-tabulations, providing preliminary insights into potential relationships between constitutional type and patients’ baseline health profiles.

A post-hoc power analysis indicated that the attained sample size (n = 60) provided approximately 80 % power to detect moderate associations between *Prakriti* classifications and Rotterdam Symptom Checklist (RSCL) domain scores at a significance level of *p* < 0.05.

The study specifically examined associations between *Deha Prakriti* types and symptom burden as measured by the RSCL, encompassing four domains: Physical Impairment, Psychological Impairment, Activity Level Impairment, and Overall Valuation of Life. Prior to analysis, data normality was assessed using the Shapiro–Wilk test. Since RSCL domain scores violated normality assumptions, non-parametric statistical methods were applied.

The Kruskal–Wallis H test was employed to compare median RSCL domain scores among the six *Deha Prakriti* groups (*Vatapradhāna–Pittanubandhi*, *Vatapradhana–Kaphanubandhi*, *Pittapradhāna–Kaphānubandhi*, *Pittapradhāna–Vatanubandhi*, *Kaphapradhāna–Vatanubandhi*, and *Kaphapradhana–Pittanubandhi*). When a significant omnibus difference was observed, Dunn's post-hoc test with Bonferroni correction was conducted to identify specific intergroup differences while controlling for multiple comparisons.

In addition, the Spearman's rank correlation coefficient **(ρ)** was calculated to assess monotonic relationships between *dosha dominance* (*vata*, *pitta*, and *kapha* percentages obtained via the AyuSoft tool) and overall RSCL total scores.

Statistical significance was set at *p* < 0.05 (two-tailed) for all analyses, and effect sizes (ε^2^) were computed for non-parametric tests to estimate the magnitude of observed associations. All statistical analyses were performed using JASP software (Version 0.18.1).

## 3 Results

### 3.1 Participants

A total of 75 patients were initially considered for participation, out of which 70 patients were assessed for eligibility. Among them, 65 patients met the eligibility criteria, and 60 patients provided consent and were included in the final analysis. As this was a cross-sectional study, there was no follow-up period, and all 60 participants were analyzed**.**

Of the 15 patients who did not participate, 5 declined due to personal reasons, while 5 were excluded due to cognitive impairment or inability to complete the Rotterdam Symptom Checklist (RSCL).

The Kruskal–Wallis analysis performed in JASP indicated no statistically significant differences in RSCL domain scores across Prakriti groups: Physical Impairment H (5) = 5.82, p = 0.213; Psychological Impairment H (5) = 3.94, p = 0.415; Activity Level Impairment H (5) = 4.51, p = 0.341; and Overall Valuation of Life H (5) = 2.77, p = 0.596.

Descriptive trends suggested higher physical and activity limitations among *vata*-dominant groups and higher psychological distress among *pitta*-dominant groups, although these did not reach statistical significance (p > 0.05 for all comparisons).

### 3.2 Descriptive data

The study included 60 cancer patients, with ages ranging from 26 to 71 years. Among them, 45 were female and 15 were male. Participants came from diverse occupational backgrounds, with homemakers being the most common**.** Geographically, the study covered 36 different cities**.**

The most common cancer types were breast cancer, ovarian cancer, and lung cancer, with some cases of colon, bladder, stomach, and neuroendocrine tumors. Several patients had metastatic cancer, indicating disease spread beyond the primary site. Some patients had missing data in these assessments. Since this was a cross-sectional study, there was no follow-up period.

### 3.3 Outcome data

The study cohort consisted of patients with different Prakriti subtypes. The majority of patients belonged to the *Kaphapradhana-Pittanubandhi* subtype, accounting for 23 patients (38.33 %). This was followed by *Kaphapradhana-Vataanubandhi*, which included 15 patients (25.00 %). The *Vatapradhana-Kaphaanubandhi* subtype comprised 9 patients (15.00 %), while both *Pittapradhana-Kaphaanubandhi* and *Vatapradhana-Pittanubandhi* subtypes had 6 patients each (10.00 %). The least represented group was *Pittapradhana-Vataanubandhi*, with only 1 patient (1.67 %).

### 3.4 Main results

*Vatapradhan-Pittaanubandhi* *prakriti* demonstrates the highest physical impairment score of 38.96, highlighting a significant burden of physical ailments. The dominance of *vata*, characterized by mobility and instability, likely contributes to this challenge. Despite this, the activity level score is the highest at 13.83, suggesting that the inherent mobility of *vata* helps maintain functionality. The psychological impairment is moderate at 8.90, reflecting occasional fluctuations in mental health due to *vata's* instability and *pitta's* fiery nature. With a lesser valuation of life, there is a slight impact on life perception, likely balanced by higher activity levels. This *prakriti* struggles with physical burdens but compensates with better functionality and mental resilience.

*Vatapradhan-Kaphaanubandhi* *prakriti* exhibits a slightly lower physical impairment score of 36.85 compared to *Vatapradhan-Pittaanubandhi*, indicating a high but somewhat mitigated physical burden due to *kapha's* stabilizing influence. However, the psychological impairment score is 12.34, showing significant mental health challenges exacerbated by *kapha's* heaviness. The activity level score is low at 8.90, likely a result of *kapha's* sluggish nature reducing functionality. The overall valuation of life is the lowest, reflecting a negative life perception stemming from compounded physical and psychological burdens. This *prakriti* faces notable challenges in physical and mental health and reduced activity, culminating in a poorer life outlook.

With a moderate physical impairment score of 30.41, *Pittapradhana-Kaphaanubandhi* *prakriti* benefits from *pitta's* sharpness and *kapha's* stability, which moderate extreme physical challenges. The psychological impairment score is the lowest at 8.81, suggesting strong mental resilience due to *kapha's* grounding effect. The activity level score is relatively high at 13.37, supported by *pitta's* drive and *kapha's* endurance. The overall valuation of life indicates a positive life outlook despite moderate physical challenges. This prakriti is characterized by balanced functionality, manageable burdens, and a good life valuation.

*Pittapradhana-Vataanubandhi* *prakriti* has the lowest physical impairment score at 24.05, suggesting excellent physical health, potentially due to *pitta's* fiery nature counteracting *vata's* instability. However, the psychological impairment score is the highest at 13.00, reflecting significant mental strain from *pitta's* intensity and *vata's* erratic influence. Despite this, the *prakriti* shows no activity level impairment, indicating the best functionality. The life valuation also reflects a highly positive outlook despite mental challenges. This prakriti exemplifies minimal physical burden and excellent life valuation but struggles with mental health.

*Kaphapradhana-Vataanubandhi* *prakriti* demonstrates a moderate physical impairment score of 27.78, slightly higher than *Pittapradhana-Vataanubandhi*, due to the stabilizing influence of *kapha* on *vata's* erratic nature. The psychological impairment score is moderate at 9.69, reflecting occasional mental challenges mitigated by *kapha's* grounding qualities. The activity level score is low at 8.95, suggesting reduced functionality stemming from *kapha's* heavy and sluggish nature. The overall valuation of life impairment is moderate, indicating a fair perception of life despite some challenges. This *prakriti* has moderate burdens and reduced activity but maintains an average life outlook.

With a moderate-to-high physical impairment score of 34.61, this *kaphapradhana-pittaanubandhi* *prakriti* experiences challenges likely arising from *kapha's* heaviness and *pitta's* sharpness. The psychological impairment score is high at 12.12, suggesting significant mental health burdens from *pitta's* intensity combined with *kapha's* natural heaviness. The activity level score is moderate at 9.39, reflecting some limitations in functionality. The overall valuation of life is moderate, indicating life perception influenced by physical and psychological challenges. This *prakriti* faces dual challenges in health but maintains average activity levels and life valuation.

Overall, *vatapradhana* types tend to exhibit higher physical burdens due to *vata's* instability but compensate with better activity levels, reflecting its inherent mobility. In contrast, *pittapradhana* types experience lower physical burdens but are more susceptible to significant psychological strain, often offset by better life valuation scores due to *pitta's* intensity and drive. Meanwhile, *kaphapradhana* types face moderate physical and psychological burdens, reduced activity levels, and average life valuations, primarily influenced by *kapha's* heaviness and stabilizing nature, which dampens mobility and mental agility.

The study, therefore, reveals that *vata*-dominant patients required more immediate intervention due to their rapid tumor growth, which was driven by the inherent *vata* qualities of volatility and sensitivity. These patients showed a heightened responsiveness to their disease, with tumors progressing at a faster pace compared to other *prakriti* types. In contrast, *pitta*-dominant patients were more susceptible to adverse effects from chemotherapy, as their *pitta*-influenced metabolism and inflammation tendencies heightened the intensity of the treatments, leading to more pronounced side effects.

Interestingly, patients with a dominant *kapha* *prakriti* displayed a slower pace of tumor progression, allowing more time for therapeutic management and intervention. This indicates that the *kapha* *prakriti* may be more resilient and adaptable to the challenges of cancer and its treatments.

Thus, the significant loss of vitality experienced by cancer patients during chemotherapy could be better mitigated through the integration of tailored Yogic practices and dietary adjustments, guided by the unique Prakriti profile of each individual. By tailoring the treatment approach to the individual's Prakriti, the study suggests that the adverse effects of cancer therapies may be better managed, and the overall well-being of the patient could be improved.

## 4 Discussion

### 4.1 Key results

The disparities observed in RSCL domains among Prakriti types corroborate the Ayurvedic principle that symptom manifestation stems from *dosha* predominance. Notably, *vata*-dominant patients exhibited elevated physical impairment, consistent with *vata* vitiation, whereas *pitta*-dominant individuals displayed heightened psychological distress, aligning with *pitta* aggravation.

The findings of this study highlight the growing significance of integrating traditional Eastern wisdom, such as *Ayurvedic* principles, with modern Western scientific approaches in the field of personalized cancer care. The study demonstrates that by considering the individual's *deha* *prakriti*, as defined by *Ayurveda*, healthcare providers can develop more targeted and effective interventions for cancer patients using yoga therapy.

This study emphasises the growing necessity of combining Ayurvedic concepts with current oncology to provide personalized cancer care. The findings show significant differences in tumour growth, therapeutic response, and resilience among *prakriti* subtypes. *vata*-dominant patients have rapid tumour growth due to innate instability and sensitivity, requiring early stabilisation treatments. *pitta*-dominant patients have more chemotherapy side effects because of their fiery and metabolic nature, demanding cooling and anti-inflammatory treatments. *kapha*-dominant patients have slower tumour growth but have issues with sluggishness and stagnation, indicating the necessity for stimulating therapy. The study further emphasises the importance of *prakriti*-based *yoga* therapy in minimizing these consequences, as it provides targeted therapies to address both the physical and psychological burdens of cancer.

### 4.2 Comparison with previous research

The observed trends in this study are generally consistent with previous research exploring Prakriti-based variability in cancer and related symptomatology [49]. Earlier studies have indicated that *vata*-dominant individuals are more prone to pain, fatigue, and variable treatment tolerance due to their physiological instability and heightened neurosensory responsiveness, whereas *pitta*-dominant types often exhibit greater psychological stress, inflammatory tendencies, and metabolic reactivity [26]. These patterns parallel our descriptive findings, wherein *vata*-predominant participants demonstrated higher physical and activity impairment scores, while *pitta*-dominant groups showed slightly elevated psychological distress.

Previous integrative oncology studies have largely focused on the molecular or genomic correlates of Prakriti, identifying associations with inflammatory gene expression, oxidative stress markers, and immune responsiveness [50]. However, few have examined patient-reported outcomes using validated instruments such as the Rotterdam Symptom Checklist (RSCL) [51]. By applying this structured symptom assessment tool, the present study contributes novel clinical evidence linking Ayurgenomic classifications with multidimensional symptom experiences in cancer patients. Although the differences observed were not statistically significant, these results align directionally with the theoretical framework of Ayurgenomics and provide a foundation for hypothesis-driven, longitudinal studies in integrative cancer care.

### 4.3 Limitations

While the study's limitations are important to acknowledge, they do not diminish the potential significance of its findings. The modest sample size, observational design, and reliance on self-reported measures represent inherent challenges in early-stage research exploring integrative frameworks. These constraints can be addressed through future, larger-scale, and controlled investigations that expand upon the foundational insights generated here.

The absence of control for certain confounding variables reflects the real-world complexity of cancer care, where multiple biological, psychological, and social factors concurrently influence outcomes. Specifically, the cross-sectional design limits the ability to account for unmeasured confounders such as psychosocial support, emotional resilience, concurrent yoga or Ayurvedic therapies, and socioeconomic background. Although participants were recruited from a uniform tertiary oncology setting and analyses were adjusted for age, sex, and cancer stage, residual confounding from these contextual factors cannot be excluded.

Importantly, this study serves as a preliminary yet meaningful step toward empirically validating the integration of Ayurvedic principles within contemporary oncology, a domain that remains underexplored. Future research should incorporate validated tools to quantify psychosocial and behavioral parameters and employ longitudinal or interventional designs to clarify how *Deha Prakriti* interacts with psychosocial and environmental determinants in shaping symptom expression and treatment outcomes. Such efforts will strengthen the evidence base for a more personalized, integrative approach to cancer care.

### 4.4 Interpretation

The study emphasises the possibility of an Ayurvedic-based approach to improving personalized cancer care. Recognising each Prakriti's distinct physiological and psychological qualities allows for targeted interventions—such as yoga therapy, dietary changes, and lifestyle modifications—to improve patient results. The findings are consistent with the ideas of integrative oncology, implying that merging traditional Eastern wisdom with Western medical advances can result in a comprehensive therapeutic paradigm. The study also emphasises the importance of early management for *vata*-dominant individuals due to their quick tumour growth, inflammation control for *pitta*-dominant patients, and activation therapy for *kapha*-dominant individuals. These findings emphasise the relevance of *dosha*-specific therapeutic techniques for increasing resilience, lowering treatment adverse effects, and improving overall quality of life.

Integrating *prakriti*-based *yogic* interventions with both conventional and modern therapies can alleviate this burden, using various *yogic* practices and dietary adjustments to restore energy and resilience. This personalized approach bridges traditional and modern care, offering a holistic pathway to manage the adverse effects of cancer and its treatments. By addressing the unique needs of each *prakriti*, Yogic interventions can not only mitigate the physical and psychological impact of the disease but also enhance the overall quality of life for cancer patients.

The analysis highlights distinct patterns of strengths and vulnerabilities inherent to each *prakriti* type, underscoring the potential value of integrating Ayurvedic frameworks with modern cancer care to offer personalized treatment strategies. The personalization of cancer care through the lens of *Ayurveda* and *Yoga* Therapy holds immense potential to improve patient outcomes, as it allows for the incorporation of holistic approaches that address the unique physiological, psychological, and emotional needs of each individual.

For individuals with *kaphapradhaana-pittaanubandhi* *prakriti*, yoga protocols should focus on stimulating circulation, reducing *kapha* stagnation, and calming *pitta*-induced hypermetabolism. A diet emphasizing light, cooling, and non-spicy foods further complements these practices by mitigating *kapha* and *pitta* imbalances.

In cases of *kaphapradhaana-vaatanubandhi*, *yoga* practices should aim to ground *vata* while activating lymphatic circulation to address *kapha*-related blockages. Guided imagery meditations and restorative postures stabilize the mind and body. Warm, nourishing, and mildly spiced foods enhance grounding and reduce *kapha* congestion.

For *pittapradhaana-kaphaanubandhi*, protocols focus on cooling and calming practices to address *pitta's* fiery nature and *kapha's* structural tendencies. Breath-awareness meditation techniques help in reducing emotional heat. A cooling, alkaline diet, avoiding sour and oily foods, complements the practice.

For those with *Vaatapradhaana-Kaphaanubandhi*, yoga should prioritize grounding *vata's* erratic energy while gently mobilizing *kapha*. Meditative practices like *japa* meditation (chanting) and restorative yoga with bolsters provide mental calm and physical ease. A diet rich in grounding and unctuous foods, such as ghee and root vegetables, enhances the benefits of the practice.

For *vaatapradhaana-pittaanubandhi*, yoga practices should balance *vata's* mobility and *pitta's* intensity. Warm, slightly unctuous foods that are not spicy or fermented aid in calming both doshas. Lastly, for *pittapradhaana-vaataanubandhi*, practices should focus on reducing *pitta's* inflammation and soothing *vata's* instability. Relaxation techniques in *shavasana* with the use of cooling essential oils provide additional relief. A diet emphasizing sweet, bitter, and astringent tastes ensures better *dosha* harmony.

### 4.5 Generalisability

While the study provides useful insights, its generalisability is limited by the small and potentially region-specific sample. The applicability of Prakriti-based cancer therapies to varied populations and ethnic groups needs further exploration. To improve generalisability, larger, multi-center research with different patient groups and rigorous clinical trials are required. However, this study is an important step towards connecting Ayurveda and modern oncology, providing a potential approach for holistic, patient-centered cancer treatment.

## 5. Conclusion

Yoga occupies a unique place in the modern management of metabolic disease. It is an ancient practice increasingly supported by scientific evidence, one that appears to influence several biological and psychological pathways simultaneously. Although their effects are moderate in magnitude, they span multiple domains of health, including metabolic regulation, stress physiology, sleep, inflammation, and autonomic balance. However, the success of Yoga as a therapeutic intervention depends heavily on factors such as long-term adherence, cultural acceptance, accessibility, and the quality of instruction and clinical training of therapy providers, which differ substantially from those of conventional pharmacological treatments.Current evidence supports the inclusion of Yoga as part of a broader strategy for managing metabolic disease. However, the methodological limitations within the existing literature also call for a balanced and careful interpretation of the findings. The most appropriate approach is therefore neither outright skepticism nor biased enthusiasm, but rather critical appraisal grounded in scientific credibility. The metabolic disease epidemic is among the defining health challenges of our era, both mortality and morbidity-wise, and its successful management will require a combination of pharmacological, behavioral, and lifestyle-based approaches. Thus, blind faith in established science is fraught with the risk of failure, and an out-of-the-box, scientific mindset may be necessary.

## Author Contributions

**Ananada Balayogi Bhavanani:** Conceptualization, Methodology, Visualization, Software, Editing, Data curation. **Nilachal Padmashanti**: Writing- Original draft preparation, Writing- Reviewing and Editing, Data curation, Software. **Uthpala Gajamanne**: Investigation, Writing- Reviewing and Editing, Software. **Vandana Srivastava***:* Methodology, Supervision. **Karthikeyan Kaliyamurthi**: Supervision, Investigation, Resources.

## Declaration of generative AI in scientific writing

In this study, a few AI tools were used like OpenAI and Julius to understand the previous published research papers in Cancer care. Also, to improve the readability and fluency of the content, AI tools were used for paraphrasing.

## Sources of Funding

This research was supported by Junior Research Fellowship awarded by the University Grants Commission (UGC), Government of India. The funding agency had no role in the design of the study, data collection, analysis, interpretation, or preparation of the manuscript.

## Conflict of interest

There is no conflict of interest.

## Data Availability

All data generated or analyzed during this study are included in this article.
